# Selective laser trabeculoplasty for anti-VEGF associated intraocular pressure elevation in steroid-naïve eyes

**DOI:** 10.3389/fopht.2026.1675048

**Published:** 2026-09-01

**Authors:** Anindya Samanta, Chelsey Krambeer, Addie Flowers, Matthew Porter

**Affiliations:** 1 Department of Ophthalmology and Visual Sciences, Texas Tech University Health Sciences Center, Lubbock, TX, United States; 2 Department of Ophthalmology, Baylor College of Medicine, Houston, TX, United States

**Keywords:** anti-vascular endothelial factor (anti-VEGF), anti-VEGF (vascular endothelial growth factor), glaucoma, selective laser trabeculoplasty (SLT), selective laser trabeculoplasty anti-VEGF

## Abstract

**Purpose:**

Selective Laser Trabeculoplasty (SLT) is utilized in cases of primary and certain secondary open-angle glaucoma to decrease intraocular pressure (IOP). SLT effectiveness has been demonstrated in patients with increased IOP secondary to intraocular steroids. In addition to intravitreal steroids, intravitreal anti-vascular endothelial growth factor (VEGF) injections are commonly used in patients with diabetic retinopathy and age-related macular degeneration. Some patients receiving anti-VEGF injections experience increases in IOP. No studies have evaluated the effectiveness of SLT for anti-VEGF-associated glaucoma.

**Methods:**

This was a retrospective, single-center study. Anti-VEGF-associated IOP increase was defined as an eye receiving at least one anti-VEGF intravitreal injection prior to SLT. IOP and the number of pressure-lowering drops were measured at 3-, 6-, 9-, and 12-month post-SLT follow-up appointments. Outcomes included changes in IOP, and the number of IOP-lowering drops prescribed.

**Results:**

Twenty-three eyes from 18 patients were included in this study. The IOP was 18.26 ± 3.33 with 2.6 ± 1. drops prescribed at baseline. Two eyes required repeat SLT. Over the study period, IOP was reduced by 3 to 4.5 mm Hg, with all p-values <0.001. Eight eyes in the study were drop-naïve (no IOP-lowering drops prescribed prior to SLT). Of these, 6/8 (75%) remained drop-free at the end of the observation period. At their final clinic visit, 78.3% of patients were controlled on SLT without further surgical or medical intervention.

**Conclusions:**

To the best of our knowledge, this is the first study to demonstrate the long-term effectiveness of SLT in anti-VEGF-associated open-angle glaucoma in steroid-naïve eyes.

## 1 Introduction

Selective Laser Trabeculoplasty (SLT) is used in primary and certain secondary open-angle glaucoma (OAG) cases to increase aqueous humor outflow and thereby decrease intraocular pressure (IOP). SLT has been shown to reduce IOP by an average of 22% at 6 months and 32% at 5 years post-SLT (1, 2). According to the LiGHT trial, SLT is an effective alternative to IOP-lowering drops in open-angle glaucoma patients (2, 3). Due to its favorable safety profile and cost effectiveness, SLT is a valid treatment option before resorting to topical drops or more invasive surgeries.

Other studies have explored SLT’s effectiveness in patients treated with intraocular steroids, which are known to increase IOP in some patients (4). Previous studies have shown the effectiveness of SLT in treating steroid-induced IOP elevation (4, 5). Tokuda et al. reported that 10 eyes with steroid-induced glaucoma showed a 35.9% reduction in preoperative IOP one year after SLT (4). Similarly, SLT has shown a comparable reduction in IOP for patients receiving one or more dexamethasone implants (6, 7). Additionally, studies have suggested SLT as a prophylactic measure against IOP elevation when applied before intravitreal steroid injections (8).

In addition to intravitreal steroids, intravitreal anti-vascular endothelial growth factor (VEGF) injections are commonly used in patients with diabetic retinopathy and age-related macular degeneration. Some patients who receive anti-VEGF injections experience sustained increases in IOP (9). Several hypotheses have been proposed to explain the sustained IOP elevation observed in some patients. One suggests that each injection damages the trabecular meshwork, causing transient increases in IOP that ultimately become sustained (10, 11). Another theory is that the injection components cause edema or direct inflammation of the trabecular meshwork (12). A third possibility is that the injection components block aqueous humor outflow, either through reduced angiogenesis or the physical properties of the injection itself (9). Finally, concerns exist about the potential for syringe or medication leaks during injections, which could inadvertently damage the eye (13–15).

Currently, the effectiveness of SLT in treating anti-VEGF-associated IOP increase remains unstudied. Limited reports and small patient cohorts suggest SLT may help manage IOP increases after anti-VEGF injections, typically following the failure of pressure control with multiple drops (16–18). No studies, whether prospective or retrospective, have systematically evaluated the effectiveness of SLT for anti-VEGF-associated glaucoma, highlighting the need for further research. This study aims to evaluate the real-world effectiveness of SLT in treating elevated IOP associated with intravitreal anti-VEGF injections.

## 2 Methods

The purpose of this study was to determine the efficacy of SLT in eyes that have and are also receiving anti-VEGF. This was regardless of the origin of IOP rise, which may be due to multiple mixed-mechanism processes. This retrospective, single-center study included eyes that met all inclusion criteria and did not meet any exclusion criteria. Anti-VEGF-associated IOP increase was defined as an eye that had received at least one anti-VEGF intravitreal injection prior to SLT. Inclusion criteria were eyes with a diagnosis of OAG or ocular hypertension that received SLT between June 2019 and June 2022 at Texas Tech University Health Sciences Center. All patients received 360 degrees of SLT treatment, with a power range of 0.6–0.8 millijoules. Only patients who continued to follow up in the glaucoma clinic were included in the study.

Exclusion criteria included a history of intravitreal steroid injections or implants, pars plana vitrectomy, and glaucoma secondary to iridocorneal endothelial syndrome, angle-closure glaucoma, inflammatory glaucoma, developmental glaucoma, or neovascular glaucoma. Selected eyes did not previously have SLT or any other anterior segment surgery, other than non-complicated cataract surgery. Additionally, patients on oral or topical steroids were also excluded from the study.

IOP and the number of pressure-lowering drops were measured at 3-, 6-, 9-, and 12-month follow-up visits. Outcomes included changes in IOP, and the number of IOP-lowering drops prescribed at those visits. IOP was measured first by the technician with a Tonopen and then confirmed with Goldman applanation by either a resident or attending physician in glaucoma clinic. The decision on whether IOP was at goal or whether additional treatment was needed was determined by a glaucoma attending (MP). Patients who missed a follow-up were not included in the analysis for that month but were eligible to be included in future follow-ups.

For statistical analysis a two-tailed, paired t-test was used to compare the IOP and the number of drops from baseline to each follow-up visit. Alpha was set to 0.05 and p-value of <0.05 was considered statistically significant. Due to inter-eye correlation, for patients with both eyes in the study, only the left eye was included in the analysis for the t-test.

Firstly, a sub-group analysis was done on drop-naïve (no IOP-lowering drops prior to SLT) eyes. The change in IOP and whether pressure lowering drops were started was measured at 3-, 6-, 9-, and 12-month follow-up visits. Secondly a sub-group analysis was done on eyes that continued to receive anti-VEGF injection after their SLT treatment. IOP and the number of pressure-lowering drops were measured at 3-, 6-, 9-, and 12-month follow-up visits.

## 3 Results

**Figure 1:**
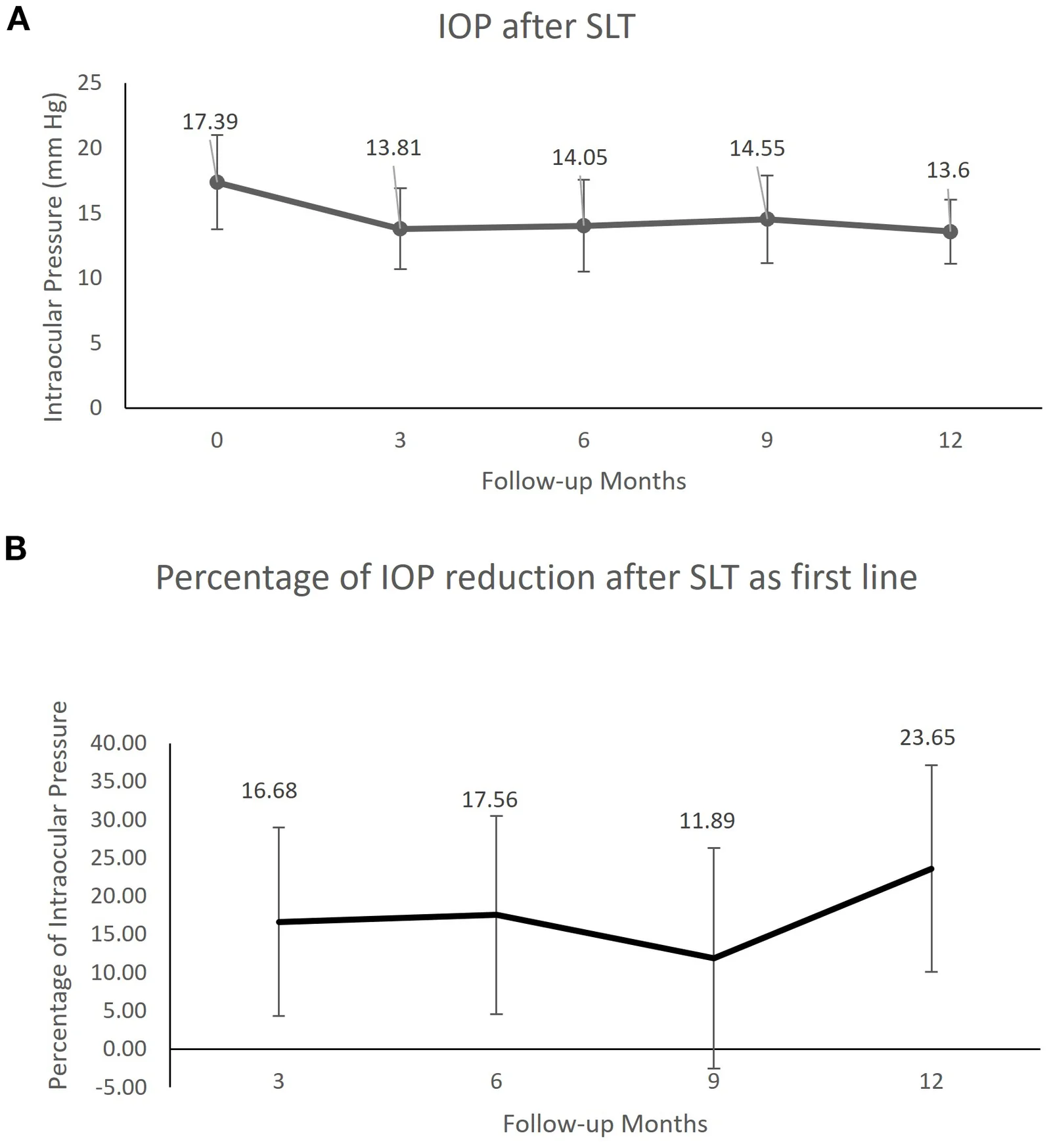
Results from eyes after undergoing selective laser trabeculoplasty (SLT). **(A)** The intraocular pressure (IOP) of eyes at 0- (baseline), 3-, 6-, 9- and 12-month visit. **(B)** The percentage of IOP reduction after SLT compared to baseline, prior to SLT treatment at 0- (baseline), 3-, 6-, 9- and 12-month visit. For **(A, B)** the graph shows the mean and the standard deviation at each time point.

Twenty-three eyes from eighteen patients were included in this study. The IOP was 18.26 ± 3.33 with 2.6 ± 1.0 IOP lowering drops prescribed at baseline. **Table 1** presents patient demographics. Seven eyes had ongoing intravitreal injections during the study, with an average of 3.0 injections after SLT. All eyes received either intravitreal bevacizumab or aflibercept in this study. **Figure 1** shows IOP results at different time points after SLT.

**Table 1:** Demographic data of patients in the study.

Demographics				Value	
Age (years)				65.8 ± 12.3	
Sex		Males		8	
		Females		10	
Race		Black		1	
		Hispanic		9	
		White		8	
Eye		Right		9	
		Left		4	
		Both		10	
IOP		Pre-SLT		18.26 ± 3.33	
IOP lowering drops		Pre-SLT		2.6 ± 1.0	
Intravitreal Injections		Pre-SLT		4.2 ± 3.3	
		To date		5.3 ± 4.2	
Follow-up (months)				10.5 ± 2.7	
POH	AMD				5
	BRVO				1
	CME from latanoprost				1
	Degenerative Myopia				1
	DR				16
Type of disease	OHTN				3
	Glaucoma		Mild		1
			Moderate		9
			Severe		10

For age, IOP, drops, intravitreal injections, and follow-up the average with standard deviation is shown. For age, IOP, IOP lowering drops, intravitreal injections and follow-up months the mean ± standard deviation is reported. Intravitreal injections and POH are shown for the eye that underwent selective laser trabeculoplasty (SLT). IOP, intraocular pressure; POH, past ocular history; AMD, age-related macular degeneration; BRVO, branch retinal vein occlusion; CME, cystoid macular edema; DR, diabetic retinopathy; OHTN, ocular hypertension.

One eye required surgical intervention (cyclophotocoagulation) after the 1-month visits and was removed from the study. This eye was using three IOP-lowering drops prior to SLT, and SLT was attempted to avoid further surgery. The patient’s other eye remained in the study. Regarding follow-up visits, 1 (5.56%), 3 (15.79%), 2 (11.11%), and 7 (36.84%) eyes did not have 3-, 6-, 9-, and 12-month follow-ups, respectively. Another eye underwent pars plana vitrectomy after the 9-month visit, unrelated to the patient’s glaucoma diagnosis, and was removed from the study.

Two eyes required repeat SLT. The first required repeat SLT at month 6 and eventually needed additional IOP-lowering drops. The second eye had repeat SLT at month 9 and remained drop-free at 1 year.

During the follow-up period, 4 eyes reduced their IOP-lowering drops by an average of 1.5 drops, while 4 eyes required an additional drop prescribed after SLT, all after at least the 6-month visit. The remaining eyes maintained their IOP-lowering drops throughout the study.

**Table 2A** shows that IOP decreased by 3 to 4.5 mm Hg over the observation period without a significant change in the number of drops prescribed compared to baseline. At 6, 9, and 12 months there was a reduction of -3.53 ± 2.87 mmHg, -2.95 ± 2.80 mmHg and -4.47 ± 3.59 mm Hg, respectively. The IOP reduction was 19.61%,15.92% and 23.90% at 6, 9 and 12 months, respectively. **Table 2B** compares both the IOP reduction and number of drops prescribed at each follow-up with only one eye included per patient. Statistical analysis shows statistically significant IOP reduction (p < 0.01) at 6- and 12-month follow-up without a statistically significant change in the number of drops prescribed (p > 0.05).

**Table 2A:** Eyes that have completed the 3-, 6-, 9- and 12- month follow up visit.

Months	Eyes	IOP (mm Hg)	Drops
		δ from baseline (mean ± SD)	δ from baseline (mean ± SD)
3	21	- 3.24 ± 2.17	- 0.14 ± 0.48
6	19	- 3.53 ± 2.87	- 0.00 ± 0.47
9	20	- 2.95 ± 2.80	- 0.10 ± 0.64
12	15	- 4.47± 3.59	- 0.20 ± 1.01

Intraocular pressure (IOP) change (δ) from baseline was calculated from pre-selective laser trabeculoplasty (SLT) IOP.

**Table 2B:** Only one eye per patient that have completed the 3-, 6-, 9- and 12- month follow up visit.

Months	Eyes	IOP (mm Hg)		Drops	
		δ from baseline (mean ± SD)	P-value	δ from baseline (mean ± SD)	P-value
3	17	- 3.24 ± 2.08		- 0.18 ± 0.52	
6	16	- 3.81 ± 2.79	<0.01	- 0.00 ± 0.52	1
9	16	- 3.50 ± 2.48		- 0.00 ± 0.65	
12	12	- 4.83 ± 2.89	<0.01	- 0.08 ± 0.90	0.75

IOP change from baseline was calculated from pre-SLT IOP A paired t-test was used to compare IOP to pre-SLT IOP where p-value < 0.05 was considered significant. Drop δ from baseline was calculated from number of drops pre-SLT. A paired t-test was used to compare drops to number of drops pre-SLT where p-value < 0.05 was considered significant. SD: standard deviation.

**Figure 2:**
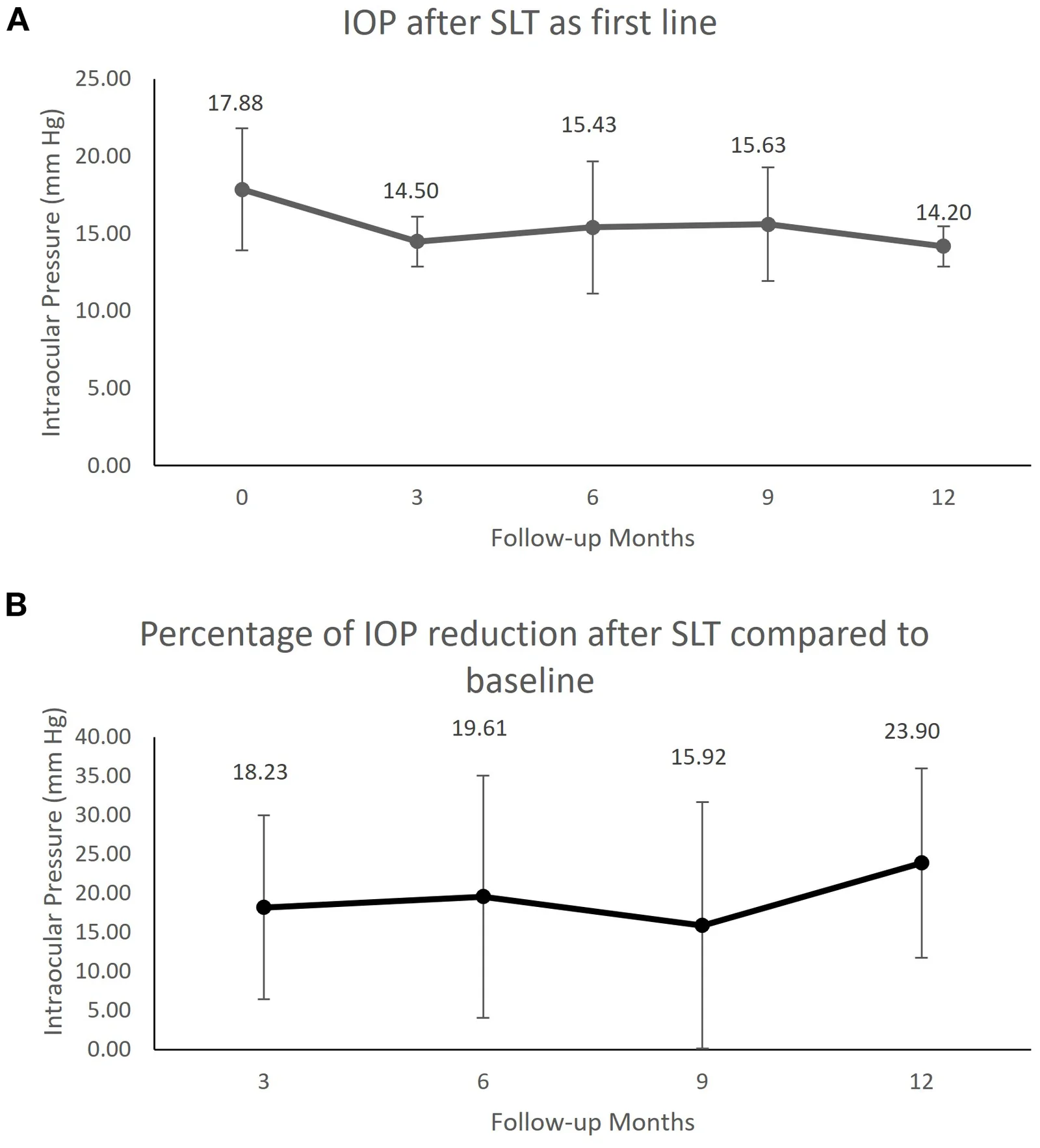
**(A)** Results from naïve eyes after undergoing selective laser trabeculoplasty (SLT) as first line therapy. The intraocular eyes pressure (IOP) of at 0-(baseline), 3-, 6-, 9- and 12-month visit. **(B)** The percentage of IOP reduction after SLT as first line therapy compared to baseline in naïve eyes, prior to SLT treatment. For **(A, B)** the graph shows the mean and the standard deviation at each time point.

In a sub-group analysis, eight eyes in the study were drop-naïve (no IOP-lowering drops prior to SLT). The baseline pre-treatment IOP in this group was 17.88 ± 3.94 mmHg. There was a reduction of 3.14 ± 2.41 (19.61%), 2.00 ± 2.43 (15.92%), and 4.80 ± 3.31 (23.90%) at 6, 9 and 12 months, respectively. Of these, 6/8 (75%) remained drop-free at the end of the observation period. The remaining 2/8 (25%) required 1 additional drop to meet IOP targets after SLT. **Figure 2** displays the results from this subgroup.

In another sub-group analysis, eyes that continued to receive anti-VEGF intravitreal injections post-SLT treatment were analyzed. **Table 3** compares the IOP changes in the 7/23 (30.4%) eyes that continued to receive anti-VEGF injections after SLT (actively treated group) with those that did not (non-actively treated group). 

**Table 3A:** Actively treated eyes were defined as eyes that continued to receive intravitreal injections post-SLT treatment.

Actively treated eyes	
	Intravitreal injections (mean ± SD)
Pre-SLT	6.1 ± 4.5
To date	9.1 ± 4.9

Non-actively treated eyes did not receive any intravitreal injections post-SLT treatment. The number of anti-VEGF injections in these eyes pre-SLT treatment and to date is shown. SLT, selective laser trabeculoplasty; SD, standard deviation.

**Table 3B:** The intraocular pressure in the actively treated group vs. non-actively treated group is shown.

Actively treated vs. non-actively treated eyes				
Months	Actively treated		Non-actively treated	
	Eyes	IOP in mm Hg (mean ± SD)	Eyes	IOP in mm Hg (mean ± SD)
0	7	17.00 ± 3.65	16	17.56 ± 3.71
3	6	12.67 ± 2.42	15	14.27 ± 3.31
6	6	12.33 ± 2.42	13	14.85 ± 3.78
9	7	14.14 ± 3.53	13	14.77 ± 3.42
12	5	12.20 ± 2.17	10	14.30 ± 2.41

A IOPs between the actively treated eyes and the non-actively treated group. Anti-vascular endothelial growth factor (anti-VEGF); IOP, intraocular pressure; SLT, selective laser trabeculoplasty; SD, standard deviation.

## 4 Discussion

This real-world study highlights the effectiveness of SLT in managing anti-VEGF-associated IOP increases. At their final recorded clinic visit, 78.3% of patients were controlled on SLT without requiring additional surgical or medical intervention.

Rubin et al. demonstrated that SLT decreased IOP by 50% in patients treated with intravitreal triamcinolone at 6 months (5). In that study, 2/7 patients required surgical intervention. Bozkurt et al. found that SLT prior to intravitreal triamcinolone injection resulted in a 1.4 mm Hg decrease in IOP at 6 months compared to untreated eyes (8). None of the SLT-treated eyes required additional IOP-lowering drops, while 8/16 of the untreated eyes did. The previous success of SLT in eyes treated with intravitreal triamcinolone are comparable to the IOP reductions observed in our study. There was a statistically significant reduction in IOP at 6, 9 and 12 months without a statistically significant increase in IOP-lowering drops (**Table 2**).

Two eyes required repeat SLT, with mixed results. One eye underwent repeat SLT at month 6 due to increased IOP and eventually needed to be started on an additional drop. A second eye underwent repeat SLT at 9 months and was able to stay drop-free for the duration of the study. Because of the small number of patients, it is unclear if there is a benefit to repeating SLT in these patients, as was observed 25% of patients in the LiGHT trial (3).

The effectiveness of SLT as a first line treatment is promising, based on the results in the drop- naïve sub-group (**Figure 2**). These eyes had not received any medical or surgical IOP lowering intervention prior to being treated with SLT. The finding that 6/8 eyes (75%) remaining drop-free after SLT, suggests that SLT may be an effective first-line treatment. The remaining 25% required only one additional drop to meet their IOP goals. Overall, there was a clinically significant decrease in this cohort at 6, 9, and 12 months.

Our study also suggests that SLT may have an effect against future IOP increases following anti-VEGF injections. Eyes that continued receiving anti-VEGF injections (actively treated group) did not show statistically significant differences in IOP compared to those that did not (non-actively treated group) in follow-up visits. Previous studies have shown that SLT may have protective role against IOP increases with future intravitreal steroids (8). Based on the results, it may serve a similar protective role against future anti-VEGF injections as well.

To our knowledge, this is the first study to demonstrate the long-term effectiveness of SLT in anti-VEGF-associated open-angle glaucoma. A strength of our study is the strict inclusion and exclusion criteria. Often intravitreal steroids and anti-VEGF are used concurrently to treat macular edema. However, by excluding eyes that had received steroids, this study attempts to examine the effectiveness of SLT in anti-VEGF associated intraocular pressure. Additionally, the consistent treatment and electronic medical records are used for all patients within a single institution, allowed accurate tracking of all anti-VEGF injections and glaucoma treatments. Another strength was the two sub-group analysis exploring SLT’s role in drop-naïve and actively treated eyes.

A limitation of this study is its retrospective design, which is subject to patient compliance and follow-up. The strict exclusion criteria, which disqualified patients with prior steroid injections, implants or surgery, do limit the sample size. Furthermore, a mix of treatment-naïve and drop-controlled patients complicates the analysis.

In conclusion, this study shows promising results regarding SLT’s effectiveness in treating anti-VEGF-associated glaucoma. This study provides valuable insight that can be further explored in prospective studies. Due to the minimally invasive and well tolerated nature of SLT and the pervasiveness of anti-VEGF intravitreal injections, SLT could potentially be considered earlier in the management of anti-VEG associated IOP increases.

## Data Availability

The raw data supporting the conclusions of this article will be made available by the authors, without undue reservation.
